# How to study deep roots—and why it matters

**DOI:** 10.3389/fpls.2013.00299

**Published:** 2013-08-13

**Authors:** Jean-Luc Maeght, Boris Rewald, Alain Pierret

**Affiliations:** ^1^Joint Research Unit Biogéochimie et Ecologie des Milieux Continentaux, IRDVientiane, Laos; ^2^Forest Ecology, Department of Forest and Soil Sciences, University of Natural Resources and Life ScienceVienna, Austria

**Keywords:** deep roots, biogeochemical and ecological functions, root measure

## Abstract

The drivers underlying the development of deep root systems, whether genetic or environmental, are poorly understood but evidence has accumulated that deep rooting could be a more widespread and important trait among plants than commonly anticipated from their share of root biomass. Even though a distinct classification of “deep roots” is missing to date, deep roots provide important functions for individual plants such as nutrient and water uptake but can also shape plant communities by hydraulic lift (HL). Subterranean fauna and microbial communities are highly influenced by resources provided in the deep rhizosphere and deep roots can influence soil pedogenesis and carbon storage.Despite recent technological advances, the study of deep roots and their rhizosphere remains inherently time-consuming, technically demanding and costly, which explains why deep roots have yet to be given the attention they deserve. While state-of-the-art technologies are promising for laboratory studies involving relatively small soil volumes, they remain of limited use for the *in situ* observation of deep roots. Thus, basic techniques such as destructive sampling or observations at transparent interfaces with the soil (e.g., root windows) which have been known and used for decades to observe roots near the soil surface, must be adapted to the specific requirements of deep root observation. In this review, we successively address major physical, biogeochemical and ecological functions of deep roots to emphasize the significance of deep roots and to illustrate the yet limited knowledge. In the second part we describe the main methodological options to observe and measure deep roots, providing researchers interested in the field of deep root/rhizosphere studies with a comprehensive overview. Addressed methodologies are: excavations, trenches and soil coring approaches, minirhizotrons (MR), access shafts, caves and mines, and indirect approaches such as tracer-based techniques.

## Introduction

Studies on below-ground ecosystem processes are relatively rare compared to those dealing with above-ground traits of plants; roots and the rhizosphere being “hidden” in the soil (Smit et al., 2000), their observation and study relies on deploying special methodologies that are generally time-consuming and often costly. Even though methodologies to study belowground processes have significantly improved and the number of studies addressing roots has increased in recent decades, studies on roots remain mostly confined to the uppermost soil horizons. While Canadell and colleagues (1996) highlighted the potential influence of “deep roots” on many ecosystem processes nearly two decades ago, information about the actual importance of deep roots in terms of plant and ecosystem functioning, (global) water cycles and biogeochemistry remains scarce. This situation appears to be related to two major factors: (i) technological and economical limitations, i.e., the absence of tools to measure roots with sufficient throughput and standardization at affordable costs (Böhm, 1979; Vogt et al., 1996; Smit et al., 2000), and (ii) the widespread assumption that deep roots are a rather marginal component of plants. Even though deep roots may, in most cases, represent a relatively small fraction of the overall root system biomass, they likely fulfill much more essential functions than commonly accepted; an increasing number of studies clearly indicate that “looking deeper” is essential to increase our understanding of plant ecophysiology, but also of community ecology and geochemical cycles (Harper and Tibbett, 2013; see below). This review highlights the increasing importance and impact of deep roots in environmental research and provide some guidance to future research.

In this context, this review elaborates on the physiological and ecological significance of deep roots before providing a detailed overview on methods to study deep roots. Addressed methodologies are (i) excavations, trenches and soil coring approaches, (ii) minirhizotrons (MRs), (iii) access shafts, (iv) caves and mines, and (v) indirect approaches such as tracer-based techniques.

## The challenge of defining deep roots and measuring rooting depths

Factors that drive root growth and root system expansion are known from a diversity of field and laboratory observations. Previous publications have described the genetic control of root traits such as length, branching and root hair formation [see references in Kell (2011)]; however, the mechanistic details, resulting in different root system phenotypes, are often unknown [but see e.g., Kato et al. (2006) for “root growth angle”]. With regard to genetic control, some root systems were found to develop rapidly: *Pinus radiata* and *Robinia pseudoacacia* roots reached a depth of 2.5 and 3.7 m after 4 years respectively (Stone and Kalisz, 1991). Similarly, Christina et al. (2011) reported that roots of Eucalypt trees could progress downward at rates of 0.55 m month^−1^, 9–10 months after planting. Beside genetics, root architecture is controlled by hormonal influences from the plant (e.g., Santner et al., 2009) and soil organisms, and by the environment.

Due to the fact that soils are the most complex of all environments (Fitter et al., 2000) and nutrients are often strongly bound to the soil matrix (Strong et al., 1999), soil resources are inherently patchy and poorly available to organisms. In turn, plants have evolved complex strategies to forage for soil resources; root growth and root system development correspond to the allocation of assimilates to individual root apices capable of independent, yet coordinated at the plant level, morphological and physiological responses to their immediate environment. In view of the major influence of soil patchiness on root growth, it is not unexpected that spatial rooting patterns are highly variable. Indeed, one major confounding factor that often precludes accurate estimation of rooting depth is the inherent variability of root distributions (e.g., Nicoullaud et al., 1995). Further, even when this variability is taken into account, sampling depths are often decided arbitrarily and set to values that are too shallow to allow reliable estimates of rooting depth (Schenk and Jackson, 2002).

However, studies focusing on rooting depth have clearly shown that woody plants are, on average, more deeply rooted than herbaceous ones (e.g., Shalyt, 1950; Baitulin, 1979; Kutschera and Lichtenegger, 1997; Schenk and Jackson, 2002). According to Canadell et al. (1996), the rooting depths of herbaceous plants, shrubs and trees are globally in the magnitude order of 2.6 ± 0.1 m, 5.1 ± 0.8 m, and 7.0 ± 1.2 m, respectively. Many trees (*Eucalyptus spp*) and shrubs in arid areas are very deep rooted, with woody legumes such as *Acacia*, and *Prosopis* reaching depths of 20 m and even extremes such as 50–60 m (Stone and Kalisz, 1991). Canadell et al. (1996) have pointed out that tropical savannah is the biome with the deepest mean rooting depth (15 ± 5 m) and also has the deepest recorded root system (i.e., 68 m; Jennings, 1974). However, even in evergreen tropical forests a number of tree species have deep root systems (>8 m), which enable them, e.g., to survive periodic droughts (see below).

Thus, aside from genetic control and the physiological needs of each single species, external physical or biochemical factors influence the root development. Indeed, Harper et al. (1991) proposed to define root system architecture (RSA) as an evolutionary response to the spatio-temporal variability of resource availability and the corresponding constraints to growth. Some studies suggested that maximum rooting depth is mostly limited by water tables or by subsoil characteristics that prevent rooting (Cannon, 1949; Stone and Kalisz, 1991; Stone and Comerford, 1994) while others demonstrated that trees can grow roots well beyond the subsoil into the weathered bedrock (Schwinning, 2010) and/or maintain active roots below the mean water table (Wardle et al., 2004; Laio et al., 2009), e.g., by carrying and releasing oxygen under water-logged conditions (Justin and Armstrong, 1987; Shimamura et al., 2007). Thus at least some plants can modify the soil properties in their immediate vicinity (Hodge et al., 2009) to allow for deeper root system placement. However, according to Schenk (2008), roots grow as shallow as possible and as deep as necessary in response to the required water supply. Despite providing a rational explanation for the development of deep roots under a range of environmental conditions, this approach overlooks other major root functions such as nutrient foraging (see below) and plant anchorage. In addition, some experiments conducted under favorable environments (with no water or nutrient constraints, no anchoring hindrances) evidenced substantial root systems (Passioura and Wetselaar, 1972), which contradicts the former statement and confirms the generic value of the concept of a plastic root growth (Hodge, 2006).

Given this inherent plastic nature of root system development and the resulting variability of rooting patterns, there is currently no consensus on the definition of “deep root.” Based on a global review of 565 root profiles, Schenk and Jackson (2002) derived average rooting profiles for 15 terrestrial biomes including all latitudes; the average of these 15 profiles indicates that soil depths of 1.1, 0.7, and 0.4 m correspond to cumulated root proportions of 95, 90, and 80%, respectively. Schenk and Jackson (2002) also found that the median sampling depth for root profiles was 0.88 m. Based on these figures, and notwithstanding species-specific or functional definitions, we therefore propose here to qualify “deep roots” in general as roots growing at soil depths of at least 1 m.

## Physical, bio-chemical, and ecological functions of deep roots

**Figure 1:**
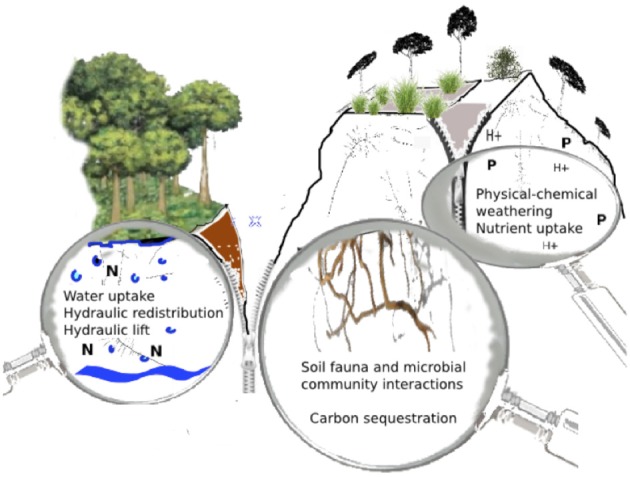
**Summary of major impacts of deep roots on the subsoil and deep roots' functions, i.e., water uptake and hydraulic redistribution, nutrient uptake, physical–chemical weathering and C sequestration, and deep root-fauna and -microbial interactions**. See text for further information.

While it is impossible to attribute most traits and functions exclusively to shallow or deep roots, some distinctions can be made in their specialization and their impact on the environment. The main ecological and geochemical impacts of deep roots are highlighted in this first part of the review and key processes are visually summarized in Figure 1.

### The role of deep roots in water uptake and redistribution

Water uptake is one of the key functions of deep root systems, especially in the driest and rockiest environments. Stone and Kalisz (1991) identified more than 30 species of trees that develop roots over long distances and can access deep water tables. Water storage in bedrock may also be of global importance: plants that experience soil moisture deficits might keep expanding their root systems in the weathered bedrock (Schwinning, 2010), an hypothesis supported by findings that shallow-soil endemic plants developed the special ability to explore large rock surface areas, which increases their chance to locate and explore cracks in the underlying rock (Poot and Lambers, 2008; Schenk, 2008). For example, evergreen forests in Northeastern Pará state in Brazilian Amazonia maintain transpiration during the up to 5-month dry periods by absorbing water from the soil to depths >8 m (Nepstad et al., 1994). Similar, most deciduous species in dry monsoon forests of South and Southeast Asia form new leaves 1–2 months before the first monsoon rains, during the hottest and driest part of the year, which indicates that climate is not the principal determinant of their vegetative phenology which most likely depends on deep rooting (Elliott et al., 2006). More surprisingly, significant contributions of deep root to plant water uptake appears not to be restricted to water-limited environments; for example, Dawson and Ehleringer (1991) found that mature riparian trees hardly used readily available stream water and derived most of their water supply from ground water at much greater depth.

It has been argued that under pronounced seasonal arid climates deep roots favor hydraulic lift (HL), also termed hydraulic redistribution (HR; Burgess et al., 1998; Burgess, 2000), i.e., the nocturnal transfer of water by roots from moist to dry regions of the soil profile. In addition to the effects on water uptake, HL and HR can indirectly influence the availability of some nutrients (Snyder et al., 2008; see below). The process of HL was probably first described by Breazeale (1930) and received much attention since the late 1980's (Richards and Caldwell, 1987; Caldwell and Richards, 1998). HL is known to predominantly-while not exclusively-occur in deep rooted vegetation of biomes such as savannahs and shrublands, mobilizing water resources down to depths of 20 m (Bleby et al., 2010). HL and HR have been reported to provide benefits for mixed species stands/intercrops in many different biomes (Peñuelas and Filella, 2003; Goldstein et al., 2008; Zapater et al., 2011) and as a consequence, to have an impact on ecosystem functioning (Horton and Hart, 1998; Oliveira et al., 2005). With regard to agro-ecosystems, HL could contribute to develop more efficient intercropping systems (Mulia and Dupraz, 2006; Malézieux et al., 2009) with positive plant–plant interactions at best acting as a “water-safety net” (Sekiya et al., 2010). Thus, it has been proposed that breeding and engineering efforts aimed at facilitating water redistribution could eventually be used to boost yields in intercropping/agroforestry systems (Burgess, 2010).

In a wider perspective, the impact of deep roots on hydrological cycles could indirectly influence regional climates; Kleidon and Heimann (2000) concluded that deep-rooted vegetation is an important part of the tropical climate system and that without considering deep roots, the present-day surface climate cannot be simulated adequately. As many tree species of tropical forests establish a link between groundwater and the atmosphere, the presence or absence of un-degraded tropical forest reportedly influences regional climate (Bruijnzeel, 2004). In summary, there is diverse, yet consistent evidence that deep roots play a major role in plant water uptake, soil water availability and the water cycle at various scales from the rhizosphere to whole catchments (Bengough, 2012)

### Deep roots and nutrient uptake

RSA, i.e., the spatial distribution and morphology of roots, root physiology and symbiotic interactions affect the ability of plants to access nutrients. The occurrence of deep-rooted plants, especially in (semi-) arid ecosystems, is classically explained in regard to water uptake (see above). However, McCulley et al. (2004) collected evidence suggesting that water uptake at depth can be limited, even under arid conditions. Furthermore, they found that some nutrients had comparable if not larger plant available pools in deeper soil layers; for example, P weathering (see below) is usually greater in deep soil layers than in the topsoil (Sverdrup et al., 2002). These results, in addition to data on strontium (Sr) uptake from deep soil horizons, suggest that deep soils in (semi-) arid regions may be more significant nutrient sources than commonly believed (He et al., 2012). In addition, HR could mobilize nutrients within the soil and supply those to roots through mass flow or diffusion (McCulley et al., 2004; Lambers et al., 2006; Da Silva et al., 2011). While data on the contribution of deep roots on nutrient uptake in other ecosystems such as highly weathered tropical soils is still scarce (Hinsinger et al., 2011), it is generally believed that deep(er) root systems are important for the uptake of mobile nutrients such as potassium (K) but also nitrogen (N). While an increase in roots length in the topsoil will not increase uptake due to overlapping depletion zones (Andrews and Newman, 1970), deep roots can significantly expand the soil volume accessible for uptake and thus, e.g., increase the N-uptake fraction (McMurtrie et al., 2012). Differences in N depletion due to differences in rooting depth are of special interest for environmental protection; N in deep soil layers is more prone to leaching than N in shallow soil horizons (Thorup-Kristensen and Nielsen, 1998; Thorup-Kristensen, 2001). While, due to the high mobility of nitrate, high root densities may not be needed to enable plants to deplete specific soil areas (Robinson, 1991; Robinson et al., 1996), a linear relationship was found between root density and ^15^N uptake from different depths (Kristensen and Thorup-Kristensen, 2004). In addition, early root growth to deeper soil horizons has been found to be important because N depletion of deep soil can be slower than N uptake in shallow soil horizons (Strebel et al., 1989), cited after (Thorup-Kristensen, 2001). For trees, Laclau et al. (2010) demonstrated that 6 m-deep roots of *Eucalyptus* spp. limited nutrient losses through deep drainage, following clear-cutting of previous tropical vegetation. While Kristensen and Thorup-Kristensen (2004, 2007) indicate that different N use efficiencies of crops depend more on species-specific differences in root development over time and space than on differences in N uptake physiology of roots, Göransson et al. (2006, 2007, 2008) found differences in the nutrient uptake capacities, i.e. root physiology, between shallow- (5 cm) and deeper-growing (50 cm) oak roots. While such differences were not found for beech and spruce, and P uptake of oak, estimates of fine root distribution alone may thus not reflect the uptake capacity of all nutrients and all tree species with sufficient accuracy (Göransson et al., 2008). Similar differences in root uptake potentials between shallow and deep roots under tropical conditions have been found for *Eucalyptus* spp. (Da Silva et al., 2011; Laclau et al., 2013). Interestingly, Pregitzer et al. (1998) found declining root respiration rates with increasing soil depth in Sugar maple. In summary, the previous studies indicate that deep rooting species such as oak, Sugar maple and *Eucalyptus* may have evolved different physiological uptake strategies in deep and shallow soil horizons, possibly optimizing uptake efficiency in terms of carbon costs by functional specialization [see also discussion in Da Silva et al. (2011)] under reduced competition. Future studies on the physiological properties of deep roots are imperative for a better understanding of the functional specialization of nutrient uptake by fine roots in general and the development of improved nutrient uptake models in specific.

### Physical–chemical weathering by deep roots

Growing roots tend to follow pores, channels and preferentially explore soil less dense than the bulk soil (Moran et al., 2000); as woody roots grow radially, they expand in volume and exert enormous pressure on the surrounding soil (Misra et al., 1986). In contrast to roots in uppermost soil horizons, growth pressure by deep roots cannot be relieved by upward displacement but by soil compaction, reducing for example, porosity and subsequently hydraulic conductivity and aeration and thus biogeochemical functioning. Even relatively consolidated, un-weathered rocks are susceptible to the physical effects of deep roots: rock wedging results when growing roots expand at joints or fractures and the pressure can accelerate chemical dissolution of minerals (Richter and Markewitz, 1995; Richter and Walthert, 2007). It has been known for decades that roots exert physical–chemical weathering actions on their environment (Meyer and Anderson, 1939), and that such processes are decisive for the mobilization of nutrients. Roots influence the ionic concentrations in their immediate environment and are also involved in other interactions due to the root exudates in the rhizosphere (Hinsinger, 1998). While such processes have almost exclusively been studied in top soils, it is certainly valid to consider that they also prevail in deep soil layers (Richter and Markewitz, 1995). Indeed, it was shown that fine roots at a soil depth of 1 m could balance chemical adversity in natural soil (Richter and Walthert, 2007). Carboxylate exudation by deep roots can contribute accessing poorly soluble iron phosphate in arid zones (He et al., 2012). As deep roots directly influence the depth distribution of soil carbon dioxide and acidity, there is no doubt that they play an active role in the physical–chemical weathering of mineral material and thus contribute to pedogenesis, but the precise biogenic effects of deep roots remain to be clarified (Richter and Markewitz, 1995).

### Influence of rooting depth on C biogeochemistry

Despite their low carbon (C) content, subsoil horizons contribute to more than half of the total soil C stocks, and therefore need to be considered in the global C cycle (Harrison et al., 2011; Koarashi et al., 2012; Harper and Tibbett, 2013). Soil organic carbon (SOC) has three main origins: plant root growth including exudates, dissolved organic carbon (DOC) transport and bioturbation (Rumpel and Kögel-Knabner, 2011). While the relative importance of each source is dependent on, for example, climate, soil and vegetation types, the general importance of roots for soil C sequestration (Kell, 2011) is underlined by the fact that the root-derived C has a high potential to be stabilized long-term. Beside other stabilizing factors (Rumpel and Kögel-Knabner, 2011), roots are often more recalcitrant than topsoil litter (Abiven et al., 2005; Rasse et al., 2005). The deposition and fate of C from deep roots (and their associated biota, see below) has rarely been examined in detail (Clemmensen et al., 2013; Harper and Tibbett, 2013). Furthermore, root C fluxes to deep soil layers are poorly understood mainly due to uncertainties associated with the measurement of total root C input, i.e., sloughing of root cells during growth, root exudates and root turnover. Because subsoil horizons with low C concentrations may not yet be saturated in SOC, it has been suggested that they may have the potential to sequester SOC through increasing C input by turnover of deep roots and DOC following preferential flow pathways such as root pores (Lorenz and Lal, 2005; Rumpel and Kögel-Knabner, 2011). The dynamics of deep SOC is largely controlled by interactions with soil minerals (Koarashi et al., 2012), and as both processes are highly influenced by deep roots (see above), future studies are urgently needed, including estimates on C changes in deep soil profiles in response to land-use changes such as de-/reforestation or the disappearance of specific deep-rooted plant species. Further studies on deep roots will significantly improve information on root-derived C, which is needed to accurately describe critical processes like net primary production and carbon storage from ecosystem to global scales and under recent and future climates (McCormack et al., 2013).

### Impact of deep roots on soil fauna and microbial communities

Fauna diversity was described as declining from the shallow toward the deep subterranean habitats (Culver and Pipan, 2009), however it is still widely unknown how deep roots influence the vertical distribution of soil fauna. While it is well known that fauna in the uppermost soil horizons and litter layers utilize roots for feed, it was also shown that deep plant roots are the major energy source, and provide shelter and cocoon-building material for troglobionts, i.e., invertebrates restricted to subterranean environments (Howarth et al., 2007; Silva et al., 2011; Novak and Perc, 2012). Both living and dead roots are used, providing resources for a wide diversity of cave organisms, including root-feeders, scavengers, and predators (Howarth, 1983). Freckman and Virginia (1989) showed that in some ecosystems the majority of nematodes, and thus herbivory, may occur at soil depths rarely studied. Because deep roots can directly or indirectly support the fauna, the loss of deep-rooted plants in general or of specific species will affect subterranean animals–as far as eliminating host root-specific animal (Reboleira et al., 2011). Knowledge on deep root-fauna interactions is thus decisive for development of conservation strategies in ecosystems and to understand root herbivory. While Silva et al. (1989) claimed that deep-rhizosphere micro-arthropod fauna is a reduced subset of the fauna of shallow soil horizons, Novak and Perc (2012) stated that the division of soil fauna into shallow and deep communities is a global pattern, at least in karst ecosystems with deep-rooted vegetation. While caves might represent very special ecosystems, the concentrations of organic matter and bioavailable nutrients usually decrease with soil depth; thus, in deep soil horizons the rhizosphere is “an oasis of resources compared with the [bulk soil]” (Richter and Walthert, 2007). For example, the fungal biomass in forest bulk soil decreased steadily by three orders of magnitude from the soil surface to 2.5 m depth whereas the fungal biomass in the rhizosphere remained relatively constant between depths of 0.4–2.5 m and was higher than in bulk soil (Richter and Walthert, 2007), illustrating the impact of roots on the depth distribution of fungal biomass. Furthermore, fungal species community compositions can change with depth too, i.e., different species or fungal functional groups form mycorrhizal symbioses with deep roots than with shallow roots (e.g., Rosling et al., 2003; Clemmensen et al., 2013). While it is known that the diversity of microorganisms is typically decreasing with depth and the community composition is changing (Eilers et al., 2012), high levels of bacterial biomass were found to remain down to 8 m depth in prairie soils (Dodds et al., 1996); it is thus currently unknown which roles deep roots play for soil microbial communities in detail. However, because deeper occurring microbes may have a greater influence on soil formation processes than their counterparts in shallow soil horizons, due to their proximity to soil parent material (Buss et al., 2005) and a critical influence on longer-term soil carbon sequestration (Rumpel and Kögel-Knabner, 2011), further studies including the rhizosphere of deep roots are imperative. A first indication of the importance of deep roots on bacterial communities is given by Snider et al. (2009), who observed complex interaction between deep roots and bacterial communities, some bacteria from the soil overlaying the cave being introduced by the roots while deep roots could acquire bacteria from the cave walls.

In general, the distributions of root-associated biota through the soil profile remains poorly understood, as most studies focus on communities in shallow soil horizons. This emphasizes the importance of future research into faunal, fungal and microbial communities adapted to the deep root zone, enhancing understanding of subterranean ecology and ecosystem functioning (Cardon and Whitbeck, 2007).

## Direct and indirect methods to study deep roots

**Figure 2:**
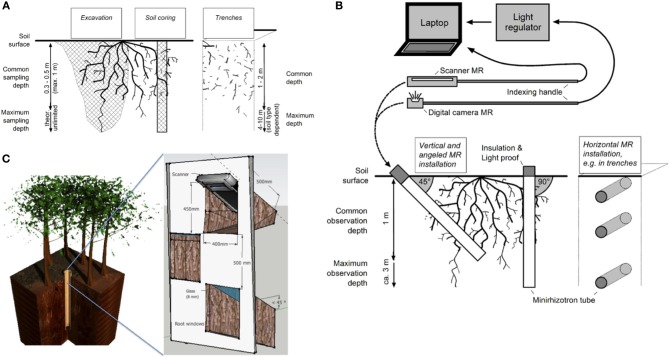
**(A–C)** Illustrations of some direct field methods to access (deep) root systems. **(A)** Excavation, soil coring and soil trenching techniques. **(B)** Minirhizotron (MR) techniques with image acquisition devices (i.e., Digital Camera or Scanner MR) and different options to install the MR tubes, i.e., angled or vertical from the soil surface or horizontally from trenches. **(C)** Schematic view of the access shafts technique. Left: Location of the well in relation to a tree row (vertical projection). Right: Side view of the soil volume excavated for angled root window installation. See text for further information.

**Figure 3:**
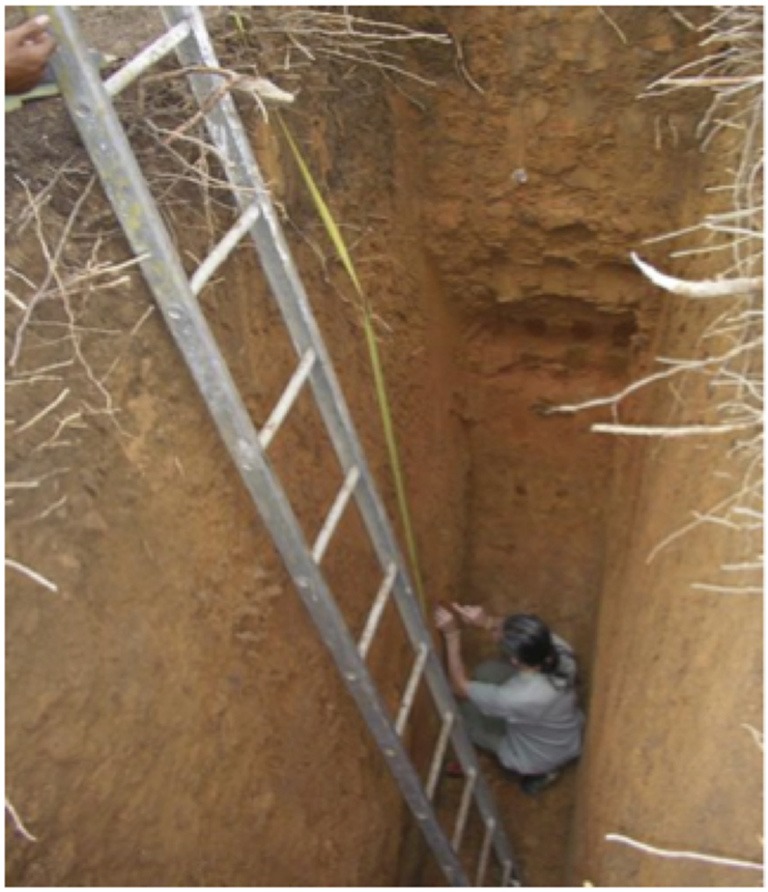
**Root mapping and collection in a trench (4 m deep) in Thailand (Maeght, 2009)**.

**Figure 4:**
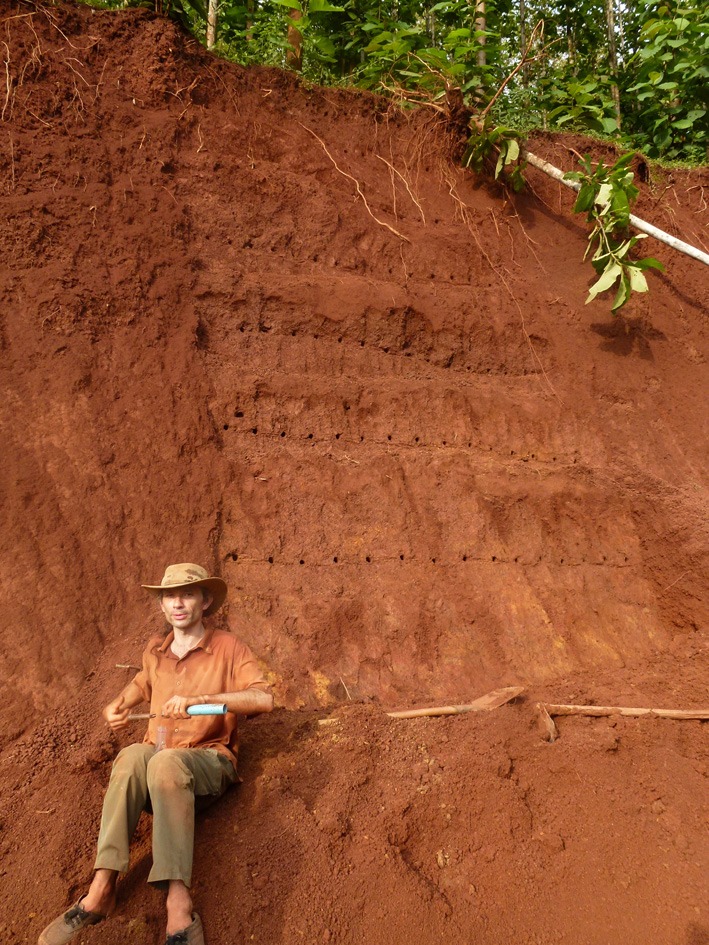
**Root sampling from an excavation (7 m deep) in Lao PDR (Maeght, 2009)**.

**Figure 5:**
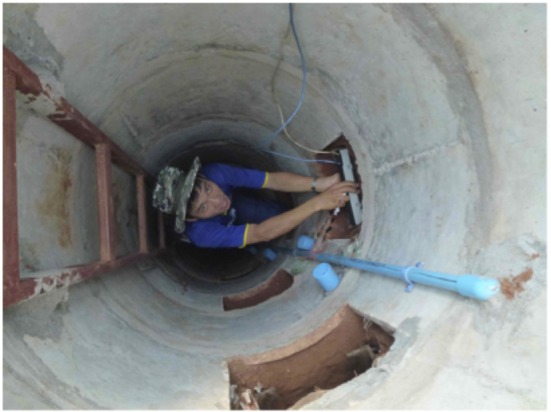
**Root scanning in access shaft (5 m deep) in Lao PDR (Maeght, 2012)**.

**Figure 6:**
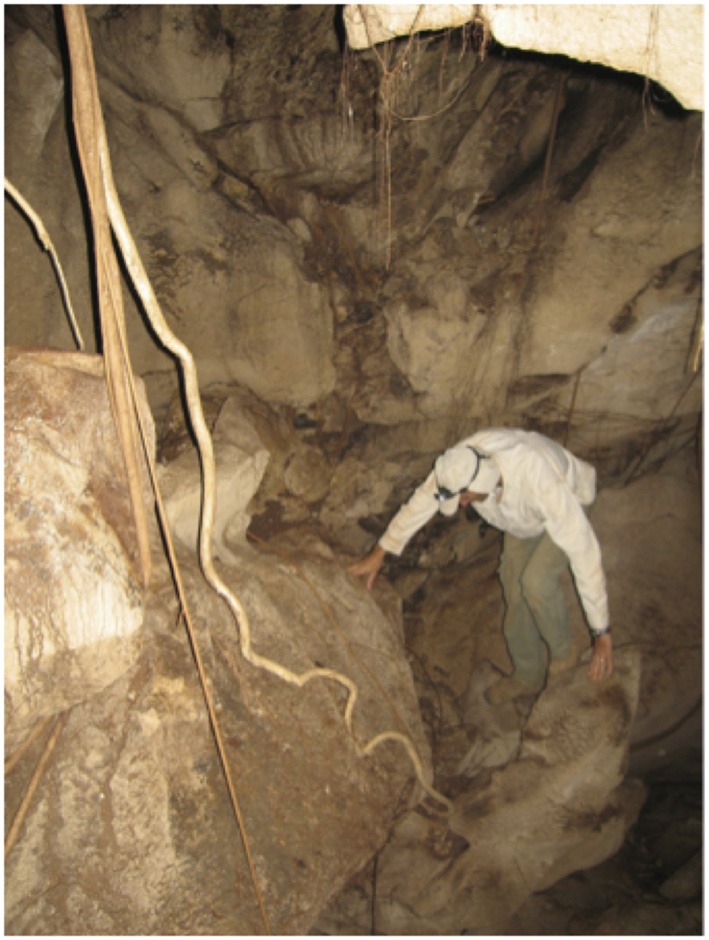
**Cave prospection (12m deep) for root studies in Lao PDR (Pierret, 2010)**.

In this second part of the review we highlight the most important methods to access and to study deep roots directly and visually (Figures 2–6) and discuss their main advantages and shortcomings (Table 1). More precisely, we present four methodological groups: (i) excavations, trenches and soil coring approaches, (ii) MRs, (iii) access shafts, and (iv) caves and mines. In addition, a short overview on (v) indirect approaches such as tracer studies is given.

**Table 1:** **Main advantages and disadvantages of direct (i.e. mechanical, visual) methods to access and to study deep roots**.

**Method**	**Realistic replication per plot**	**Key benefits**	**Disadvantages**
Excavation	Very few (~1–2)	3D information, possibility of mapping root systems layer by layer (root biomass). Root samples can be analysed further (e.g. for root morphology, to digitize the coarse root system)	Fine roots are often omitted. Very destructive and very labor intensive for bigger plants
Trenching	Few (<3)	Vertical and horizontal information (2D, root counting). Possibility to take root and soil samples and to install MR tubes and other measurement gear	Difficulty to establish deep trenches without reinforcements. Limited time of usability. Destructive and labor intensive
Soil coring	Many (>10–20)	Vertical information (fine root biomass). Root samples can be analysed further (e.g. for root morphology). Easy to replicate in stone-free soils. Minor plot disturbance	Requires a large number of samples. Moderate destructive and labor intensive rinsing. Logistically difficult if machine drilled
Minirhizotrons	Average (5–8)	Continuous, vertical information (fine root length density, root dynamics). Relatively easy to replicate in stone-free soils. Minor plot disturbance	Difficult set-up into deep soil layers (“gap formation”). Time lag before first measurement. Limited length of commercial tubes (<3 m). Expensive imaging equipment. Very labor intensive analysis and logistically difficult if machine drilled
Access shafts	Few (<3)	Continuous, vertical information (fine root length density, root dynamics). Possibility to manipulate/sample roots and soil at different depths. Sufficient space for additional measurements/devices	Adaptation depends on soil type and local geography. Moderate plot disturbance and very labor intensive. Logistically difficult for enforcement delivery
Mines and caves	Not controllable	Can provide cost-efficient access to the greatest depth. Intrinsic potential to study root-cave animal/microbe interactions. Sufficient space to install (sap-flow) sensors	Not a “normal” soil environment. Difficulties in identifying the parent plant taxa/individual from the root. Replication not controllable. Often difficult to enter

*Description of key benefits is based on one replicate per method*.

### Excavation, trenches, and coring approaches

Despite advances in root studies in the last five decades, the most common methods used to obtain data on root distribution and structure have not changed substantially: excavation and coring techniques are still and by far the preferred methods. Recently, the term “shovelomics” was establish (Trachsel et al., 2010) to qualify simple but effective approaches to determine root phenotypes including maximum rooting depth. Excavation methods include manual digging and up-rooting, the use of various mechanical devices, explosives, and high pressure water or air (Weaver, 1919; Stoeckeler and Kluender, 1938; Mitchell and Black, 1968; Newton and Zedaker, 1981; Rizzo and Gross, 2000). Coring can be conducted manually by pushing or hammering sampling equipment into the soil using various devices from simple, sharpened steel augers to advanced cryogenic devices for sampling wetland soil (Cahoon et al., 1996; Rewald and Leuschner, 2009). In addition, vehicle-mounted or hand-held mechanical devices have been developed to take soil cores in the field, especially to greater depth or with larger diameters (see Kornecki et al., 2008 and references within). An overview on the historical use of coring and excavation methods for root studies can be found in Böhm (1979).

While commonly used, most excavation and trenching approaches (Figures 2A, 3, 4) are limited to the first meter and reach only occasionally soil depths of two meters and below (Wearver, 1915; Eamus et al., 2002; Silva and Rego, 2003; Dauer et al., 2009; De Azevedo et al., 2011). While commercial trench diggers, e.g., for sewer placement, can easily be used to excavate at greater soil depth (e.g., 5 m), the stability of unsupported side walls, which depends on soil type and moisture levels (Vanapalli and Oh, 2012), is the major obstacle limiting pit/trench depth. However, occasionally several meters deep trenches can be established (Figure 3). The cost of establishing deep trenches lead many researchers to use available soil profile-walls, created by road cuts, exposed at stream cut-banks or after landslides, to determine vertical rooting pattern (Canadell et al., 1999; Silva and Rego, 2003). Common analyses at all profile-walls are root counts and estimationsL of the root length density RLD; “trench profile” technique (Van Noordwijk et al., 2000) and the determination of maximum rooting depth. While some innovations such as radiotracers (Abbott and Fraley, 1991; see below) or digital imaging (Dauer et al., 2009) have been introduced, overall profile-walls are used to quantify roots by soil location in a similar manner since the end of the 19th century (Weaver and Bruner, 1927 and references within). In contrast, excavations (Figure 2A) give full biomass per individual and often allow taking photographs/3D-scans of whole (coarse) root systems (Wagner et al., 2010)–providing valuable data on the vertical and horizontal root system distribution. However, because excavations, especially of larger plants, are particularly labor intensive, they are frequently restricted to the analysis of the upper soil layers, omitting deep roots of mature plants, and/or to low sample numbers (Cameron, 1963; Silva and Rego, 2003; Fang et al., 2012).

Soil coring approaches (Figure 2A) are suitable to obtain estimates of root length and mass, and root morphology beside data on root distribution. However, root coring is also often restricted to the uppermost soil layers because the majority of fine roots can be found in the first 0.3–0.5 m of soil. In addition, the occurrence of stones or boulders or high soil densities can prevent the use of simple and cheap manual coring tools for sampling of deep roots. However, corers have occasionally been taken to a much greater soil depth with technical help; for example Virginia et al. (1986) took samples down to the water table at 5–6 m depth in the Sonoran Desert, and Ritson and Sochacki (2003) sampled roots down to six meters with a motor driven corer to determine the root biomass of *Pinus pinaster* in Australia. Rarely much greater soil depths are explored by machine drilling of cores (<20 m, Carbon et al., 1980; <34 m, Dalpé et al., 2000). At moderate depths, soil coring was found to be a more efficient option for fine root distribution mapping than trenching (Dauer et al., 2009) but this advantage might not hold for deeper soil horizons. Upscaling from core data to stand level root biomasses is in general only possible if sample numbers are sufficiently high due to heterogeneous root distribution (see above). For deep roots this might be especially problematic because of the low biomass of deep roots and their even more heterogeneous distribution; thus, high sample numbers are essential for deep root sampling by soil coring (Bengough et al., 2000).

### Minirhizotron techniques

Non-destructive methods for studying root systems, rhizotrons, “root windows” and MRs have the advantage of allowing the repeated observation of particular locations in the soil profile. The techniques also permits visualization of very small roots, and occasionally hyphae, through the transparent observation windows/tubes. The MR method was probably first used by Bates (1937); Bates, and described again later (Waddington, 1971; Vos and Groenwold, 1983; see Rewald and Ephrath, 2013 for a recent review). This method is now widely used in multiple fields of root research, such as studies on root distribution and root demography, and interaction between roots and root-soil (organisms) (Poelman et al., 1996; Majdi et al., 2005). Setting up MR tubes in the field requires the use of a soil corer (Hummel et al., 1989) or manual auger (Kage et al., 2000), and can be technically complex depending on the nature of the soil (e.g., smearing of walls with high clay content, presence of gravels preventing progress, lack of cohesiveness in sandy soils or in water saturated soils, etc.). Nevertheless, some researchers have successfully installed MR tubes in rocky soil (Phillips et al., 2000) and in wetlands (Iversen et al., 2011). MR tube installation from the soil surface (vertical or angled; Figure 2B) rarely occurs beyond the first meter of the soil profile, due to the above-mentioned difficulties encountered during installation (Rewald and Ephrath, 2013). For soil with higher bulk densities and to access greater depths, researchers need to use portable mechanical drilling devices or tractor-mounted auger systems (Brown and Upchurch, 1987; Kloeppel and Gower, 1995). Furthermore, the length of commercially available transparent observation tubes (norm: 2 m long, max. length: approx. 3 m) presents a constraint for continuous tube installation to greater depth. This problem is partially circumvented by researchers by installing MR tubes horizontally in rhizo-lysimeters or from trenches (Figure 2B). However, because of the workload such attempts have been extremely rare; examples are the field-based rhizo-lysimeter complex of Charles Sturt University, Australia (Eberbach et al., 2013) and MR tube installation in 8 m deep trenches in a plantation of eucalypt trees in Brazil (Hinsinger et al., 2012).

The MR method permits calculation of fine-root length production, mortality and turnover (Trumbore and Gaudinski, 2003); the same fine-root segments can be monitored over their lifetime and pictures are stored in a database for processing (Rewald and Ephrath, 2013). However, the conversion of MR data, i.e., RLD, to root biomass requires the simultaneous collection of root cores to develop correlations. Compared to excavated roots and repeated coring approaches, the MR technique allows relatively continuous segregation of live and dead root since image sequences that span the life-time of roots are acquired (but see Rewald and Ephrath, 2013). However, it has been documented that one major limitation of MR studies with regard to the assessment of root turnover is that they over-sample the smaller and more dynamic lower-order roots (Guo et al., 2008).

A common limitation of the MR technique (Johnson et al., 2001) is the difficulty in obtaining good contact between the tube and the soil; in many soil types gaps form in some places along the tube, creating artificial conditions for root growth. This problem is suggested to aggravate with increasing drilling depth (“off-centered”) and the use of machine drilling which creates less precisely sized holes than manual hammering. In conclusion, MR tubes installed from the soil surface rarely reach much more down than one meter because this is the depth to which manual installation is often possible. The installation of deep horizontal MR tubes, e.g., in trench profiles, is difficult due the limited space for using an auger and inserting tubes, and laborious due to the additional trenching. However, the most serious limitations to the MR technique seem to be the initial costs of hard- and software and the time lag until soil and root dynamics come back to steady state conditions after tube installation. Furthermore, while labor costs for tube installation and picture capturing are relatively moderate, image analysis can become very time consuming and sufficient resources have to be scheduled for these purposes.

### Access shafts

The access shaft (or access well) observation technique (Figures 2C, 5) is a recent evolution and combination of the different techniques for root observation described in Böhm (1979) and in the two previous method sections of this review. The access well method provides safe access to deep soil observation locations, by means of ladders affixed to the well's wall. Depths of several meters, typically between 5 and 10 m depending on soil conditions, can be investigated. Building the well can take about a week and the walls are reinforced with concrete tubes or other materials (Maeght et al., 2012), distinguishing this techniques from trenches. Importantly, wells maximize the accessible soil depth: volume of displaced soil ratio, compared to other types of excavations.

Similar to MR techniques, access shafts allow direct observation of root growth dynamics using adapted “root windows” through which roots can be observed at regular time intervals. Using an access-well and a window scanner technique, following a procedure similar to that described by Maeght et al. (2007), root growth dynamics and root turnover could be monitored at 0.5 m soil depths increments down to 4.5 m in a rubber tree plantation in NE Thailand (Gonkhamdee et al., 2009). The number of root windows should be adapted to the well depth; windows should be geometrically arranged to allow for complete observation of the profile without compromising the strength of the reinforcing structure. Each root window includes a specifically designed glass frame supporting, on its upper side, a piece of 10 mm thick glass (~25 × 30 cm) pressed against the soil at a 45° angle (Figure 2C; Maeght et al., 2012). On the frame's lower side, two guide rails allow the insertion of a standard flatbed scanner; the images can be analysed analogue to pictures from MR tubes and similar constrains to data analysis apply (see above). However, the advantage of the access shafts method is that it provides physical access to deep soil horizons for (manipulative) research, e.g., to measure microbiological activities, and nutrient and water uptake *in situ*. Access shafts also allow the installation of various sensors at soil depths that have not been investigated in greater detail, examples are special devices for imaging the dynamics of soil pH as influenced by roots (e.g., optodes, Blossfeld et al., 2011) or NIR/VIS portable spectrometry analysis (Nakaji et al., 2008).

### Caves and mines

Deep roots of trees and shrubs are regularly found in caves and mine shafts (Cannon, 1960, cited after Stone and Kalisz, 1991; Stone, 2010). However, such observations have most often been mentioned in the literature as curiosities. Only in the last decade caves have been used more systematically for studies on roots. In 1999, Jackson et al. used 21 different deep caves (5–65 m deep) in the Edwards Plateau, USA to study the community composition below ground and maximum fine root depth of six dominant tree species (Jackson et al., 1999). They linked deep roots to each species and individual DNA sequence variation of the internal transcribed spacer (ITS) and inter-simple sequence repeats (ISSR) (Rewald et al., 2012), and found that all six tree species grew roots below 5 m, and at least four of the six reached a depth of 18 m. Similarly, Howarth et al. (2007) determined species composition of deep roots in Hawaiian lava tube caves with DNA sequence variation and related root taxa to cave arthropod fauna. In more recent years, the caves utilized by Jackson et al. (1999) were frequently used for further studies, e.g., to compare the hydraulic parameters of deep vs. shallow roots and to determine the water flux thru deep roots (McElrone et al., 2004, 2007; Bleby et al., 2010). In Europe, Filella and Peñuelas (2003) studied tree access to deep water sources and the possibility of HL from the deep roots of one *Pinus nigra* tree. They enriched the deep roots with deuterium by accessing them from a cave at 8 m depth, showing that, in this Mediterranean forest and during the dry summer, *P. nigra* trees accessed a deep water source and recycled it via HL. In Australia, Doody and Benyon (2011) installed sap-flow sensors on *P. radiata* roots, extending through a limestone cave to an unconfined aquifer 14 m below the surface, to quantify the contribution of deep roots to whole plant water uptake (>22%). Thus, caves can provide access to intact, functioning deep roots and several research groups have taken advantage of these natural access tunnels to deep roots in the past. While research in caves of mesic areas has been conducted (e.g., McElrone et al., 2004; Novak and Perc, 2012), results of root-specific studies are overwhelmingly available for deep roots in (karst) caves of (semi-) arid ecosystems. Aside from questions of maximum rooting depth and species community composition below ground, research mainly addressed root hydraulics and water flux patterns *in situ*. The abundance of caves and the unique environment of caves are two factors limiting the broad use of this technique, especially for studies of deep root functioning in “normal” soil environments and for quantifying deep roots.

### Indirect approaches for the observation deep roots

Quite a few indirect approaches have been used to study and quantify the role of deep roots in plant species and on the environment; while this is outside the focus of this review we will give an overview on some of them in the following.

To assess differences in uptake capacity between different soil depths, *tracers* can be injected at different depths for later recovery in the biomass; the amount of tracer in plant biomass is related to the uptake from each depth (Lewis and Burgy, 1964). Tracer element can be either radioactive or stable isotopes, or analogous elements. Analogous are chemical elements, which are similar to specific nutrient ions, thus uptake, and integration into biomass works the same way as the nutrient (e.g., Sr^2+^ instead of Ca^2+^). Some factors must be considered to successfully use tracers: (i) the application method must label the respective soil horizon uniformly and dilution effects must be predictable, (ii) the root-available amount of tracer must be predictable with respect to competing processes such as microbial immobilization and soil adsorption, and (iii) the uptake capacity of the tracer by roots, compared to (other) nutrients (i.e., discrimination factor), should be known under different soil properties (after Göransson et al., 2006, modified).

*Electrical capacitance* has been proposed as a means to estimate root mass based on the premise that the equivalent parallel resistance-capacitance of the electrical circuit formed by the interface between soil water and plant root surfaces is proportional to the overall amount of active roots present. Good correlations between root capacitance and root mass were obtained for young plants (Chloupek et al., 2006). However, the relative influence of deep vs. shallow roots on root electrical capacitance remains unclear (Herrera et al., 2012).

*Electrical resistivity tomography* (ERT) can be used to monitor soil water movement in large volumes of soil. A field study with 3-month-old maize showed that this technique could be used to non-destructively quantify in 2-D, root water uptake as well as preferential infiltration and drainage under plant rows (Michot, 2003). More recently, ERT was used as part of an experiment set up in a mature tropical forest in eastern Amazonia to demonstrate greater depletion of soil water in the 11–18 m depth increment of a throughfall exclusion plot compared with a control in the experiment (Davidson et al., 2011). These authors used a soil water content measure obtained with a TDR probe to convert soil apparent electrical resistivity values to soil water contents. Despite its sensitivity to soil characteristics, which can affect its performance, ERT is an effective means to obtain, non-destructive, indirect information about root functioning at considerable soil depths.

*Soil moisture measurements*, assessing soil moisture changes over time, represent an indirect way to detect signs of root activity namely water uptake. For example, based on soil moisture measurements, Calder et al. (1997) found clear evidence of water uptake down to a soil depth of 7.5 m under three species of plantation trees. Based on an analysis of water balance changes in a crop sequence with lucerne, Dunin et al. (2001) estimated an apparent root extension for lucerne 2–2.5 m beyond that of annual crops. Similar, simple rainfall and groundwater monitoring can be used to relate the survivorship/transpiration of some species in arid systems to the plant's ability to tap water from permanent water tables, which are sometimes located at depths of 18 m or more (Rawitscher, 1948).

## Conclusion and outlook

Although the literature does not include, by far, as many references on deep roots as it does on shallow roots, the available information has clearly demonstrated that deep roots are common and of pivotal importance for plant functioning, subterranean biocenosis and many biogeochemical cycles and associated ecosystem services such as pedogenesis, soil carbon sequestration and moisture regulation in the lower troposphere. We hope that this review will lead to a sustained interest on deep roots and the deep rhizosphere in the future; while it remains difficult to define “deep roots” in an absolute manner, there is a pressing need to reassess current root sampling and monitoring schemes, to avoid introducing bias in future assessments of root system traits. Because no methodologies exist today to characterize the entire RSA of mature plants at once, particularly not for large-sized organisms such as trees, the methods presented in this review need to be improved further. Clever combinations of techniques, such as access shafts, must be developed toward reaching deeper soil horizons at lower costs—allowing for more frequent “deep-root”-studies. While we predict that research on deep roots and the deep rhizosphere will remain laborious in the years to come, the crucial knowledge gained in regard to plant and ecosystem functioning by “looking deeper” will leave us no choice, especially not in times of increasing climate change.

### Conflict of interest statement

The authors declare that the research was conducted in the absence of any commercial or financial relationships that could be construed as a potential conflict of interest.
